# Group B Streptococcus Colonization among Pregnant Women Attending Antenatal Care at Tertiary Hospital in Rural Southwestern Uganda

**DOI:** 10.1155/2016/3816184

**Published:** 2016-05-22

**Authors:** Abdul Namugongo, Joel Bazira, Yarine Fajardot, Ngonzi Joseph

**Affiliations:** ^1^Department of Obstetrics and Gynecology, Mbarara University of Science and Technology, Mbarara, Uganda; ^2^Department of Microbiology and Parasitology, Mbarara University of Science and Technology, Mbarara, Uganda

## Abstract

*Objectives. *This study sought to determine the prevalence and factors associated with group B streptococcal anogenital colonization among pregnant women attending antenatal care at Mbarara Regional Referral Hospital, a tertiary hospital.* Methods.* Cross-sectional study where 309 pregnant women ≥ thirty-five weeks of gestation attending antenatal clinic were consecutively recruited between January and March 2015. Anovaginal swabs were collected and tested qualitatively using rapid visual immunoassay GBS test kits for presence of GBS antigens. Data was analyzed using STATA version 12. In univariate analysis, GBS colonized mothers were presented as percentages and numbers, and in multivariate analysis logistic regression analysis was applied to determine the associations of exposure variable and GBS colonization; a value of less than 0.05 was considered significant.* Results.* Mothers' median age was 25 years, 14.6% mothers being obese. GBS prevalence was 28.8%, 95% CI: 23.7–33.9. Obesity was the only significant factor associated with anogenital GBS colonization with odds ratio of 3.78, 95% CI: 1.78–8.35, a *p* value of 0.001. Maternal ages, educational level, residence, and gravidity were not associated with GBS anogenital colonization.* Conclusion.* Group B streptococcal anogenital colonization among pregnant women attending antenatal care at tertiary hospital, in Southwestern Uganda, is high.

## 1. Introduction

Group B Streptococcus (GBS) anogenital colonization is a major risk factor to early neonatal sepsis worldwide [1]. Worldwide, GBS colonization varies between 12 and 27% [2]; however this prevalence varies from place to place [3, 4], meaning we cannot rely on prevalence of a neighbouring country or continent to estimate the prevalence in our setting. Specific sites in sub-Saharan Africa revealed contradicting prevalence [5, 6].

No study in Uganda has documented GBS anogenital prevalence and factors associated with GBS anogenital colonization among pregnant women and yet in Mbarara Regional Referral Hospital (MRRH), according to records for January to May 2014, 63% of all neonatal admissions on pediatrics ward were from maternity ward of MRRH. Based on Kiwanuka et al., 2013 [7], in Mbarara Regional Hospital, Mbarara, Uganda, one of 26 neonate positive blood cultures contained GBS. Neonates acquire GBS infections from their mothers during the process of childbirth [8]. Intrapartum antibiotic prophylaxis reduces the risk of a neonate acquiring GBS infection from the mother during the process of childbirth [9].

Knowing the prevalence will help inform the clinicians whether there is a need for anogenital GBS colonization screening of pregnant women attending antenatal clinic, while identifying factors associated with its colonization will lead to targeted screening of high risk pregnant women using minimum resources available, all of which will hopefully contribute to reduction of cases of neonatal sepsis caused by GBS infection at MRRH.

At present in Uganda, there is no policy for routine GBS screening of pregnant women attending antenatal care; therefore no treatment is offered to those affected, and yet if a policy is formulated and effected, this would contribute to prevention of 2 to 3 per 1000 live births who get serious GBS neonatal infection with increased mortality and morbidity [10].

Therefore, this study aimed at determining the prevalence and factors associated with GBS colonization among pregnant women attending antenatal care at MRRH.

## 2. Methods

This was a cross-sectional study among pregnant women at ≥35 weeks of gestation, attending antenatal clinic at Mbarara Regional Referral Hospital conducted over a period of 3 months between January and March 2015. Mothers who were at ≥35 weeks of gestation and had consented to participate were included while those who had been on antibiotics treatment within the last two weeks prior to study were excluded.

### 2.1. Sampling Procedure

Participants were recruited using a consecutive sampling technique until the sample size was achieved. The principal investigator reviewed the antenatal cards of the antenatal clients as they came to the observation area for blood pressure and weight measurements. Using each participant's last normal menstruation period, weeks of gestation were calculated using Naegele's formula. Those at ≥35 weeks of gestation had an informed consent sought; those willing to participate in the study then signed or thumbprinted on the consent form. A pretested questionnaire was then administered, physical examination was performed, and anogenital specimen was collected using a Dacron swab.

Participant's sociodemographic data, history of current pregnancy, previous miscarriages, preterm labor, and stillbirths data were gathered. A general physical examination, obstetrical examination, collection of study samples, and completion of the routine ANC visit for that day followed.

The swabs were collected as follows; after additional counseling prior to collection of anogenital samples, the principal investigator wore a pair of latex gloves and, in the presence of a female nurse as a chaperone, asked the mother to lie in the dorsal position. While at the foot of the bed the study principal investigator, with the help of a research assistant, would examine the external genitalia and vaginal introitus, after separation of the labia. One sealed sterile swab was used to swab the lower vagina (without speculum placement) and a second sealed sterile swab was used to swab the anal canal. The swabs were then labeled and immediately processed in the clinic by the principal investigator with the assistance of a research assistant according to the manufacturer's instructions (Safecare Biotech, (Hangzhou) Co., Ltd.)

Specimens, reagents, and/or controls were processed at room temperature (15–30°C):

- A clean extraction tube was placed in the designated area of the workstation. Four drops of reagent A were then added to the extraction tube, and then 4 drops of reagent B would follow, and the contents mixed by gentle swirling of the extraction tube.
- Immediately both the anal and vaginal swabs would then be immersed into the extraction tube. A circular motion was used to roll the swab against the side of the extraction tube, to allow the liquid to be expressed from the swab and reabsorbed.
- The swabs immersed in the extraction tube were left for 10–15 minutes at room temperature, before they were firmly squeezed against the tube to expel as much liquid as possible. The extraction tube was then capped with the attached dropper tip and the swabs were discarded, according to guidelines for the handling of infectious agents.

The rapid strep B test kit was removed from its sealed pouch and placed on a clean level surface. The device was labeled at this point with patient or control identification. The assay was performed within 10 minutes of swab collection.

Three drops (approximately 120 *μ*L) of extracted solution from the extraction tube were added to the sample well on the test device. Trapping air bubbles in the specimen well (S) was avoided, and no solution was added to the observation window.

After ten minutes of waiting for the appearance of colored band(s), results were read and interpreted as follows:

 For a positive result, two colored bands appeared on the membrane: one band in the control region (C) and another band in the test region (T).
 For a negative result, only one colored band appeared in the control region (C) with no apparent colored band in the test region (T).
 For an invalid result, this occurred if a test did not produce a control band after 15 minutes of waiting. This kit would then be discarded; then the sample remaining in the extraction tube would be used onto a new kit.

Mothers were given their results 20 minutes after picking their anogenital swabs and those who tested positive for GBS were counseled about the result and indicated on their antenatal care card to act as notification to their primary care obstetricians.

Those who tested GBS negative were given their results and discharged from the study.

To avoid double recruitment a GBS+ signature was put onto the antenatal care card of the client indicating that the client was a participant in the GBS study.

Results were stored by the principal investigator with limited access for other personnel.

### 2.2. Data Collection

Data was collected using a pretested, coded questionnaire to gather sociodemographic and other relevant history data and findings on physical examination. The antenatal profiles, such as HIV status, were collected from patients' files/antenatal cards while results of anogenital specimens were obtained from the laboratory request forms. Data collection tools were initially piloted on 50 participants and adjustments were made accordingly in consultation with the study team. These were not included in final analysis. Data was collected by the principal investigator and trained research assistants.

The dependent variable was maternal GBS anogenital colonization at MRRH while independent variables were constituted by information collected on socio demographics, obstetrical factors like gravidity, history of early neonatal febrile illness or death, prolonged rupture of membranes, preterm delivery in the previous pregnancies, and other factors like HIV serostatus, BMI (calculated as weight of the mother in kilograms divided by her height in meters squared), and history of herbal medicine use during the current pregnancy.

### 2.3. Data Entry and Analysis

Data was entered and cleaned using Epi Info version 7, analyzed using STATA version 12, where in univariate analysis GBS colonized mothers were presented as percentages and numbers and in multivariate analysis logistic regression analysis was applied to determine the associations of exposure variable and GBS colonization, and a value of less than 0.05 was considered significant.

### 2.4. Quality Control

A pretested questionnaire was used to collect data.

Every 20th test kit was read by a laboratory technologist from the Department of Microbiology, MUST, who was not part of the study.

### 2.5. Ethical Considerations

We obtained informed consent and the study was approved by Mbarara University of Science and Technology institutional review board.

## 3. Results

### 3.1. A Total of 309 Participants Were Recruited

As shown in Table 1, most participants (40%) were of the age range 21–25 years, from urban areas (68%), married (92.6%), and HIV negative (92.6%). 14.6% were obese.

**Figure 1:**
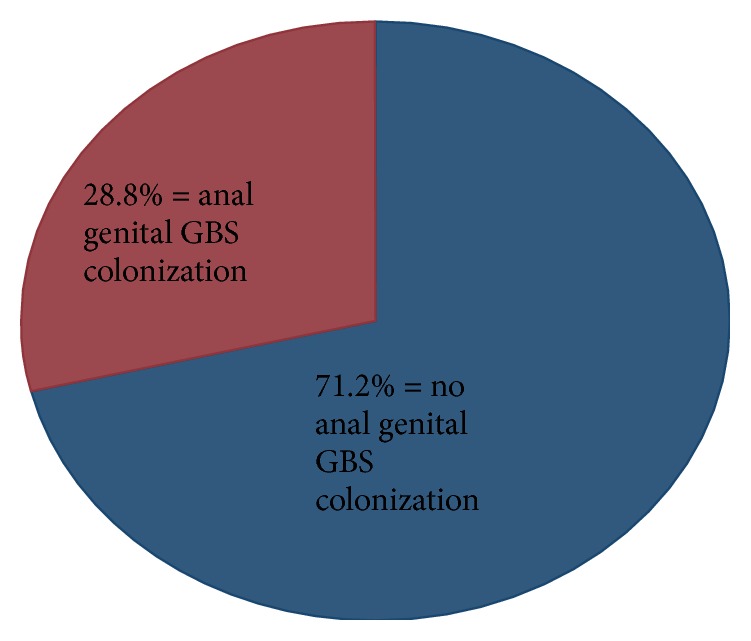
GBS anogenital colonization.

As shown in Figure 1, the GBS anogenital colonization was 28.8%.

Majority (72.8%) of the participants had had more than one pregnancy. Approximately 7.8% of the participants reported history of stillbirths. The majority of women tested for GBS (54.4%) were between 35 and 37 completed weeks of amenorrhea (Table 2).

Although there was a 2.3 odds' ratio of being anogenitally colonized by GBS among participants with perineal tears (Table 3), there was no significant association between having perineal tears and GBS colonization. Only a BMI ≥30 was significantly associated with GBS colonization (Table 4).

## 4. Discussion

The prevalence of GBS colonization among antenatal mothers in this study was 28.8%. This high prevalence means the number of neonates at a risk of developing GBS associated infection is high, hence the need for screening all mothers attending antenatal care and prophylactically treating all those colonized by GBS bacteria.

This study's high GBS anogenital colonization prevalence is comparable to findings in other parts of the world, that is, in Kenya, in a study by Mohamed 2009 [11] who at Kenyatta National Hospital found a prevalence of 25.2%. Were et al. [12], in another Kenyan study, identified a prevalence of vagina and anorectal colonization by GBS among pregnant women at term using PCR, of 30.7% at Moi Teaching and Referral Hospital (MTRH), Eldoret. In Tanzania, Joachim et al., 2009, at Muhimbili National Hospital in Dar es Salaam found a maternal anogenital colonization prevalence of 23%. In Europe, Barcaite et al., 2008 [3], found different but comparable prevalence in Eastern Europe, Scandinavia, and Southern Europe of 19.7–29.3%, 24.3–36%, and 6.5–32%, respectively. However in Tanzania a study (maternal anogenital GBS and listeria monocytogenes colonization among pregnant women) by Ernest et al. [13], conducted at a tertiary hospital in Mwanza, found a low prevalence of 9.49%, which could have been a result of the nonselective blood agar used where other organisms could have outgrown GBS.

Kiwanuka et al. [7] study at Mbarara hospital did not find any GBS colonized mother, at the hospital where this study was conducted; this could be due to the fact that they only did vaginal swabs after delivery leaving out anorectal swabs, and yet, during labor and delivery vaginal examinations, antiseptics are used and then mothers are discharged on antibiotic prophylaxis after delivery.

In Maputo, Mozambique, Steenwinkel et al., 2008 [6], found a prevalence of 1.8% which is very low compared to this study at MRRH. Possible explanation for this difference could be personal hygiene habits of thorough genitalia washing with soap and water following use of the toilet, which could have influenced the flora of the urogenital tract [6].

This study recruited pregnant women from various areas in western Uganda both rural and urban places giving us a good representation of the general population and, because mothers were taken through the procedure of screening during counseling, they believed the results as none objected to them at the time of results release.

### 4.1. Body Mass Index

Abnormal body mass index was found to be associated with GBS anogenital colonization of pregnant mothers. Pregnant mothers who were obese (BMI ≥ 30) were four times more likely to be colonized with GBS in their anogenital areas compared to mothers who were nonobese. The underlying aetiology of the association between GBS colonization and obesity that we identified is not clear; little is known about the biologic mechanism of colonization, which consequently limits its interpretation. However, it may be related to changes in the gastrointestinal microbial ecology with obesity. Animal and human studies demonstrate a shift towards increased Firmicutes (the phylum to which GBS belongs) and decreased* Bacteroides* with obesity as quoted by Ley et al. [14]. These shifts reflect increased energy-reabsorbing potential of different ratios of Firmicutes and* Bacteroides*, especially in the digestion of fatty acids and dietary polysaccharides. In addition, probably poor perineal hygiene may also contribute to GBS colonization, whereby the participant's size may prevent them from thorough anogenital cleaning as suggested by Steenwinkel et al., 2008 [6].

This finding of GBS being associated to obesity was similar to Shah et al., 2011 [15], who conducted a retrospective double cohort study at San Francisco General Hospital, California, between 2007 and 1997 and found out that obesity was one of the factors that are significantly associated with GBS rectovaginal colonization.

Several other studies have linked obesity to GBS colonization in both pregnant and nonpregnant women [15–17]. However, Najmi et al., 2013 [4], who conducted a hospital based study in Aga Khan Hospital, Karachi, had findings that were contrary to the results of our study. They concluded that GBS colonization is inversely proportional to increase in BMI; no scientific explanation is given for this finding. However, the confidence interval signified a possibility of no association.

This study's strengths include comprehensive data being compiled by principle investigator and trained research nurses that enabled us to perform detailed analysis controlling for confounders. Further, by including only women at ≥35 weeks, we focused on the subset of women in whom additional risk factors would influence clinical management if GBS status is unknown.

## 5. Conclusion

In conclusion, the prevalence of GBS colonization among pregnant mothers attending antenatal care at MRRH is high and maternal obesity was the only significant factor associated with GBS anogenital colonization among mothers attending antenatal care at MRRH.

## Figures and Tables

**Table 1:** Participants' sociodemographic characteristics.

Variable	Number (%)
*Age group (years)*	
<21	61 (19.7)
21–25	124 (40.1)
26–30	82 (26.5)
31–35	23 (7.4)
>35	19 (6.2)
*Education level*	
No education	8 (2.6)
Primary	106 (34.3)
Secondary	120 (38.8)
Tertiary	75 (24.3)
*Residence type*	
Rural	99 (32)
Urban	210 (68)
*Marital status*	
Married	286 (92.6)
Not married	23 (7.4)
*Tribe*	
Banyankore	233 (75.4)
Bakiga	30 (9.7)
Baganda	38 (12.3)
Others	8 (2.6)
*BMI*	
18.5–24.9	107 (34.6)
25–29.9	157 (50.8)
≥30	45 (14.6)
*Occupation*	
No employment	71 (23.0)
Peasant farmer	72 (23.3)
Professional	33 (10.7)
Self-employed	121 (39.2)
Casual labor	12 (3.9)
*HIV serostatus*	
Positive	23 (7.4)
Negative	286 (92.6)
*Religion*	
Muslim	42 (13.6)
Catholic	105 (34.0)
Protestant	126 (40.8)
Born again	32 (10.4)
Others	4 (1.3)

**Table 2:** Participants' obstetric and clinical characteristics.

Variable	*N* = 309	GBS positive = 89	GBS negative = 220
*Gravidity*			
Primipara	115 (37.2)	26 (29.2)	89 (40.5)
Multipara	155 (50.2)	50 (56.2)	105 (47.7)
Grand multipara	39 (12.6)	13 (14.6)	26 (11.8)
*Previous pregnancies history*			
Prelabor rupture of membranes	14 (4.5)	3 (3.4)	11 (5)
Fever during labor	38 (12.3)	10 (11.2)	28 (12.7)
Perineal tear	45 (14.6)	19 (21.3)	26 (11.8)
Preterm birth	12 (3.9)	3 (3.3)	9 (4.1)
Stillbirth	24 (7.8)	4 (4.5)	20 (9.1)
Early neonatal death	11 (3.6)	1 (1.1)	10 (4.5)
Abortion	38 (12.8)	10 (11.2)	28 (12.7)
Ectopic	5 (1.6)	0 (0)	5 (2.3)
*Current pregnancy*			
WOA			
35–37.6	168 (54.4)	48 (53.9)	120 (54.5)
38–40.6	114 (36.9)	36 (40.4)	78 (35.5)
≥41	27 (8.7)	5 (5.6)	22 (10)
LAP	116 (37.5)	30 (33.7)	86 (39.1)
Herbal medicine use	240 (77.7)	71 (79.8)	160 (72.7)
Dysuria	109 (35.3)	17 (19.1)	48 (21.8)
Abnormal pervaginal discharge	92 (29.8)	26 (21.2)	66 (30)
Herbal administration			
Vaginal	27 (8.7)	6 (6.7)	21 (9.5)
Oral	214 (69.3)	66 (74.1)	148 (67.2)

**Table 3:** Association between GBS colonization status and potential sociodemographic factors.

Bivariate analysis					Multivariate analysis	
Variable	GBS positive = 89	GBS negative = 220	COR 95% CI	*p* value	COR 95% CI	*p* value
Age group (years)				0.003		
<21	13	47	1			
21–25	26	99	0.95 (0.45–2.01)		0.8 (0.37–1.73)	0.57
26–30	31	51	2.19 (1.03–4.69)		1.81 (0.82–4.00)	0.14
31–35	8	15	1.93 (0.67–5.54)		1.15 (0.36–3.69)	0.81
>35	8	11	4.97 (1.66–14.91)		2.39 (0.69–8.17)	0.17
Education level				0.65		
No education	3	5	1			
Primary	26	80	0.54 (0.12–2.42)			
Secondary	36	84	0.71 (0.16–3.14)			
Tertiary	24	51	0.78 (0.17–3.45)			
Residence type				0.04		
Rural	21	78	1			
Urban	68	142	1.78 (1.01–3.12)		1.60 (0.88–2.9)	0.12
Marital status				0.76		
Not married	6	17	1			
Married	83	203	1.16 (0.44–3.04)			
Tribe						
Others	4	4	1	0.48		
Banyankore	63	170	0.37			
Bakiga	9	21	0.43			
Baganda	13	25	0.52			
BMI				0.0000		
18.5–24.9	24	84	1			
25–29.9	37	117	1.06 (0.59–1.89)		1.05 (0.58–1.9)	0.87
≥30	27	19	4.77 (2.28–9.98)		3.78 (1.78–8.35)	0.001
Occupation				0.74		
No employment	18	53	1			
Peasant farmer	19	53	1.06 (0.–2.2)			
Professional	9	24	1.1 (0.43–2.8)			
Self-employed	38	83	1.34 (0.7–2.6)			
Casual labor	5	7	2.1 (0.59–7.4)			
HIV serostatus				0.12		
Positive	10	13	1			
Negative	79	207	2.02 (0.85–4.78)			
Religion				0.32		
Others	1	3	1			
Muslim	17	25	2.04 (0.2–21.3)			
Catholic	24	81	0.89 (0.9–8.9)			
Protestant	38	88	1.3 (0.13–12.86)			
Born again	9	2	1.17 (0.11–12.82)			

**Table 4:** Association between GBS colonization status and potential obstetric risk factors.

Bivariate analysis					Multivariate analysis	
Variable	GBS positive = 89	GBS negative = 220	COR 95% CI	*p* value	COR 95% CI	*p* value
*Gravidity*						
Primigravida	26 (29.2)	89 (40.5)	1	0.17		
Multigravida	50 (56.2)	105 (47.7)	1.63 (0.94–2.83)			
Grand multigravida	13 (14.6)	26 (11.8)	1.71 (0.77–3.79)			
*Previous pregnancies history*						
Prelabor ROM	3 (3.4)	11 (5)	0.66 (0.18–2.43)	0.54		
Perineal tear	19 (21.3)	26 (11.8)	2.03 (1.06–3.88)	0.03	1.46 (0.66–3.21)	0.35
Preterm birth	3 (3.3)	9 (4.1)	0.82 (0.22–3.09)	0.77		
Stillbirth	4 (4.5)	20 (9.1)	0.47 (0.16–1.42)	0.18		
Early neonatal death	1 (1.1)	10 (4.5)	0.99 (0.37–2.63)	0.98		
Abortion	10 (11.2)	28 (12.7)	0.87 (0.40–1.87)	0.72		
Ectopic	0 (0)	5 (2.3)	—	—		
*Current pregnancy*						
WOA				0.36		
35–37.6	48 (53.9)	120 (54.5)	1			
38–40.6	36 (40.4)	78 (35.5)	1.18 (0.70–1.98)			
≥41	5 (5.6)	22 (10)	0.57 (0.21–1.59)			
LAP	30 (33.7)	86 (39.1)	0.79 (0.48–1.33)	0.38		
Herbal medicine use	71 (79.8)	160 (72.7)	1.19 (0.65–2.18)	0.57		
